# Improving transgene expression and CRISPR‐Cas9 efficiency with molecular engineering‐based molecules

**DOI:** 10.1002/ctm2.194

**Published:** 2020-10-04

**Authors:** Hengji Zhan, Aolin Li, Zhiming Cai, Weiren Huang, Yuchen Liu

**Affiliations:** ^1^ Guangdong Key Laboratory of Systems Biology and Synthetic Biology for Urogenital Tumors, Institute of Translational Medicine Shenzhen Second People's Hospital, The First Affiliated Hospital of Shenzhen University Shenzhen China; ^2^ Department of Urology Shenzhen Second People's Hospital, The First Affiliated Hospital of Shenzhen University, International Cancer Center Shenzhen University School of Medicine Shenzhen China

**Keywords:** CRISPR‐Cas9, innate immune response, mammalian cells, transgene expression

## Abstract

As a novel and robust gene‐editing tool, the Clustered Regularly Interspaced Short Palindromic Repeats CRISPR‐associated protein 9 (CRISPR‐Cas9) system has revolutionized gene therapy. Plasmid vector delivery is the most commonly used method for integrating the CRISPR‐Cas9 system into cells. However, such foreign cytosolic DNAs trigger an innate immune response (IIR) within cells, which can hinder gene editing by inhibiting transgene expression. Although some small molecules have been shown to avoid the action of IIR on plasmids, they only work on a single target and may also affect cell viability. A genetic approach that works at a comprehensive level for manipulating IIR is still lacking. Here, we designed and constructed several artificial nucleic acid molecules (ANAMs), which are combinations of aptamers binding to two key players of IIR (β‐catenin and NF‐κB). ANAMs strongly inhibited the IIR in cells, thus improving transgene expression. We also used ANAMs to improve the gene‐editing efficiency of the CRISPR‐Cas9 system and its derivatives, thus enhancing the apoptosis of cancer cells induced by CRISPR‐Cas9. ANAMs can be valuable tools for improving transgene expression and gene editing in mammalian cells.

## Graphical abstract

**Graphical Headlights**

1. Artificial nucleic acid molecules (ANAMs) are combinations of aptamers binding to the key players of the innate immune response (IIR).
2. ANAMs strongly inhibited the IIR in cells, thus improving transgene expression.
3. ANAMs improved the gene editing & regulation efficiency of the CRISPR‐Cas9 system.

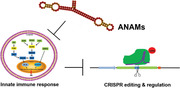

## 1 INTRODUCTION

The Clustered Regularly Interspaced Short Palindromic Repeats CRISPR‐associated protein 9 (CRISPR‐Cas9) system is a novel and robust gene‐editing tool, which has revolutionized gene therapy. The most commonly used CRISPR‐Cas9 system was isolated from the *Streptococcus pyogenes* where it is used to protect the bacteria from invasion by DNA molecules.^1^ This effective RNA‐guided gene‐editing system consists of Cas9 nuclease and two RNAs, bound to each other to form a partially paired duplex to complete Cas9 nuclease.^2^ During practical applications, the bound RNAs are shortened and combined to construct a complete single‐guide RNA, which results in a more concise and effective version than the one found naturally in cells.^3^, ^4^ Many plasmid vectors exist with constitutive promoters, which drive high expression of both components of the CRISPR‐Cas9 in the targeted host cells.^3^, ^5^, ^6^, ^7^ Although these vectors improve the efficiency of the CRISPR‐Cas9 system in eukaryotic cells, they can still be attacked by the innate immune response (IIR) within cells as exogenous substances, reducing the gene‐editing efficiency.

As the cell's first line of defense against infection, the IIR is composed of several enzymes and pathways that protect cells against damage from foreign DNA material such as infection by bacteria or viruses.^8^, ^9^, ^10^ In mammalian cells, where DNA is restricted to the nucleus, the cytosolic DNA is thought to signify infection.^11^ Once the cytosolic DNAs are detected by the IIR, this event triggers a series of changes in the cellular cascade of kinases, leading to the activation of transcription factors that induce the expression of type I interferons (IFNs) and inflammatory cytokines.^11^, ^12^ In the presence of IFNs and the inflammatory cytokines, the downstream genes are activated within cells, which cause cellular apoptosis, inhibit the translation of proteins, or induce other mechanisms to cope with the invasion of pathogens.^13^ However, this defense mechanism, while producing defense against foreign pathogens, severely hinders the efficiency of CRISPR‐Cas9 within mammalian cells.^14^, ^15^ In most cases, the necessary components of CRISPR‐Cas9 are constructed into plasmid vectors, but when the plasmid DNA appears in the cytoplasm, it activates the IIR within cells and inhibits the expression of CRISPR‐Cas9.^16^, ^17^, ^18^

Host pattern‐recognition receptors (PRRs) recognize the conserved components of invading microbes and activate the IIR within cells via type I IFNs.^19^ There are several PRR pathways in mammalian cells, which are responsible for sensing cytosolic DNAs.^20^ Previous studies have attempted to improve transgene expression by suppressing the effect of IIR on plasmid expression via inhibiting the PRRs. For example, removal of CpG motifs in plasmids successfully prevented the action of Toll‐like Receptor 9 (TLR9), which reduced the effect of IIR on plasmids and thus improved transgene expression in mice.^21^ In another study, it has been shown that the small molecule inhibitor, BX‐795, increased lentiviral transgene expression in natural killer cells (NK92)^22^ by suppressing TBK‐1 kinase in the PRR pathway. Also, the silencing of interferon receptors with short hairpin RNAs (shRNAs) could enhance the expression of the transgene.^23^, ^24^ These studies demonstrated that inhibition of the IIR system improves transgene expression to some degree in mammalian cells. However, there are deficiencies in these systems. For example, the specific inhibition of a single PRR might not adequately inhibit the IIR. Besides, the use of shRNAs to suppress PRR might cause damage to cells due to potential off‐target effects, which may hinder future applications in vivo.^25^

In the present study, we designed and constructed “artificial nucleic acid molecules” (ANAMs), which suppressed the rejection by the IIR system of plasmid DNAs simultaneously from different directions, thus increasing the efficiency of transgene expression. This new technology consisted of a special single‐stranded RNA molecule, which could specifically bind to the proteins it recognized and suppress their function. The ANAMs we described here could inhibit the function of different types of IIR‐related proteins simultaneously. Taken together, the use of ANAMs improved the efficiency of transgene expression and increased the efficiency of gene editing using the CRISPR‐Cas9 system.

## 2 RESULTS

### 2.1 Design and construction of ANAMs

**FIGURE 1:**
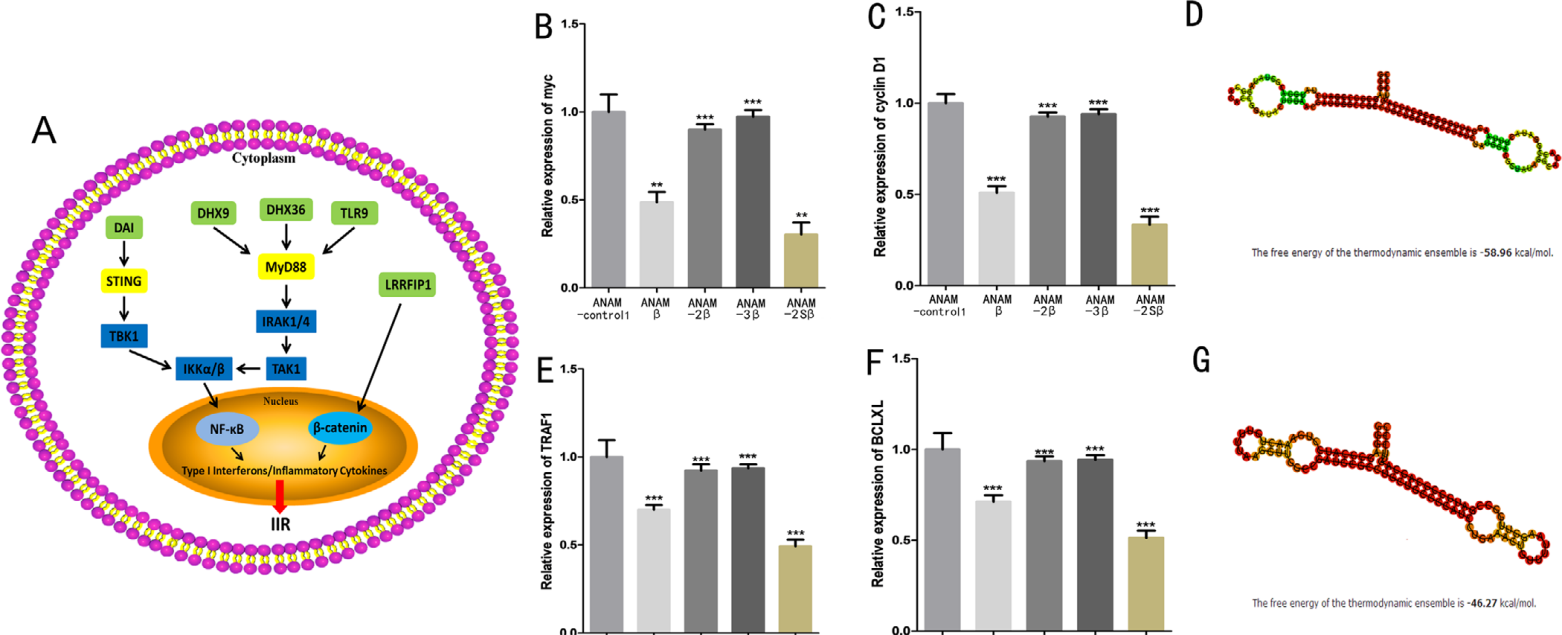
Innate immune response to cytosolic DNA and the inhibitory effects of ANAMs on targeted molecules in 5637 cells. A, DNA sensors (green) activate the downstream kinases (yellow) once they bind to cytosolic DNA. The transcription factors (β‐catenin, NF‐κB) are activated by a series of phosphorylation events, translocate to the nucleus, and induce the expression of type 1 interferon and inflammatory cytokines, which are then secreted and transmit innate immune responses to other cells. B, qPCR was used to evaluate the inhibitory effects of ANAMs on β‐catenin and NF‐κB. Inhibitory effects of ANAM‐β, ANAM‐2β, ANAM‐3β, and ANAM‐2Sβ on the expression of myc in 5637 cells. C, Inhibitory effects of ANAM‐β, ANAM‐2β, ANAM‐3β, and ANAM‐2Sβ on the expression of cyclin D1 in 5637 cells. D, Secondary structure and nucleic acid sequence of ANAM‐2Sβ. The structure was colored by base‐pairing probabilities. Pale colors indicate that a base‐pair cannot be formed in some sequences of the alignment. E, Inhibitory effects of ANAM‐κB, ANAM‐2κB, ANAM‐3κB, and ANAM‐2SκB on the expression of TRAF1 in 5637 cells. F, Inhibitory effects of ANAM‐κB, ANAM‐2κB, ANAM‐3κB, and ANAM‐2SκB on the expression of Bcl2X in 5637 cells. G, Secondary structure and nucleic acid sequence of ANAM‐2SκB. All experiments were repeated three times. ^**^ *P* < .05, ^***^ *P* < .001 compared to the ANAM control group

PRRs (DAI, DHX9, DHX36, and TLR9) in the cytoplasm rapidly recognize double‐stranded DNAs (dsDNA) and activate the IIR system, which plays an important role in interfering with plasmid expression (Figure 1A). Previous studies had demonstrated that the production of type I IFNs and inflammatory cytokines was achieved via the upregulation of transcription factor NF‐κB after the PRRs sense the dsDNAs.^13^ Also, the host pattern‐recognition receptor LRRFIP1 was reported to mediate the production of type 1 IFN and inflammatory cytokines via the β‐catenin dependent pathway.^26^ As displayed in Figure 1A, the inhibition of plasmid DNA expression by IIR is mainly accomplished through β‐catenin and NF‐κB‐dependent pathways (Figure 1A). We hypothesize that simultaneous blocking of binding of the β‐catenin and NF‐κB transcription factors to their downstream targets reduces the inhibition of plasmid expression by the IIR, thereby increasing plasmid transgene expression.

Therefore, we designed and constructed ANAMs in an attempt to prevent the interaction between β‐catenin and NF‐κB and their downstream targets. These engineered ANAMs were composed of one or two tandemly arrayed cDNA copies of previously characterized protein‐binding RNA aptamers for β‐catenin or NF‐κB and were inserted downstream of U6 promoter.^27^ First, we constructed ANAM‐β with one aptamer of β‐catenin and drove its expression with the U6 promoter. β‐Catenin interacts with DNA‐bound TCF family proteins to activate transcription of target genes, such as cyclin D1 and c‐myc.^28^, ^29^ We, therefore, tested the inhibitory effect of ANAM‐β on β‐catenin by measuring the expression levels of cyclin D1 and c‐myc in the bladder cancer cell line 5637, which displayed a high expression level of β‐catenin and NF‐κB and a moderate transgene expression efficiency in our previous studies.^30^, ^31^ We found that the expression levels of cyclin D1 and c‐myc were suppressed by approximately twofolds when ANAM‐β was present (Figure 1B,C).

Furthermore, to investigate whether the array of multiple aptamers of β‐catenin effectively improved the inhibitory effect of ANAM on β‐catenin, two or three β‐catenin aptamers were connected to construct ANAM‐2β and ANAM‐3β. Surprisingly, both aptamers not only could not improve the inhibitory effect on β‐catenin compared to the ANAM‐β, but also could not significantly inhibit β‐catenin (Figure 1B,C). We speculated that there might be crosstalk between the arrayed β‐catenin aptamers and each aptamer could not be folded to form the unique structure necessary for β‐catenin binding. To address the stability problem of the multi‐hairpin aptamers, we constructed an RNA scaffold and inserted it between the two β‐catenin aptamers to stabilize the multi‐hairpin β‐catenin aptamers (Figure 1D). We expressed these two artificial RNA elements, ANAM‐β and ANAM‐2Sβ in 5637 cells, and evaluated their inhibitory effects on β‐catenin. As compared to ANAM‐β, ANAM‐2Sβ led to about 70% reduction effects on β‐catenin (Figure 1B,C).

**FIGURE 2:**
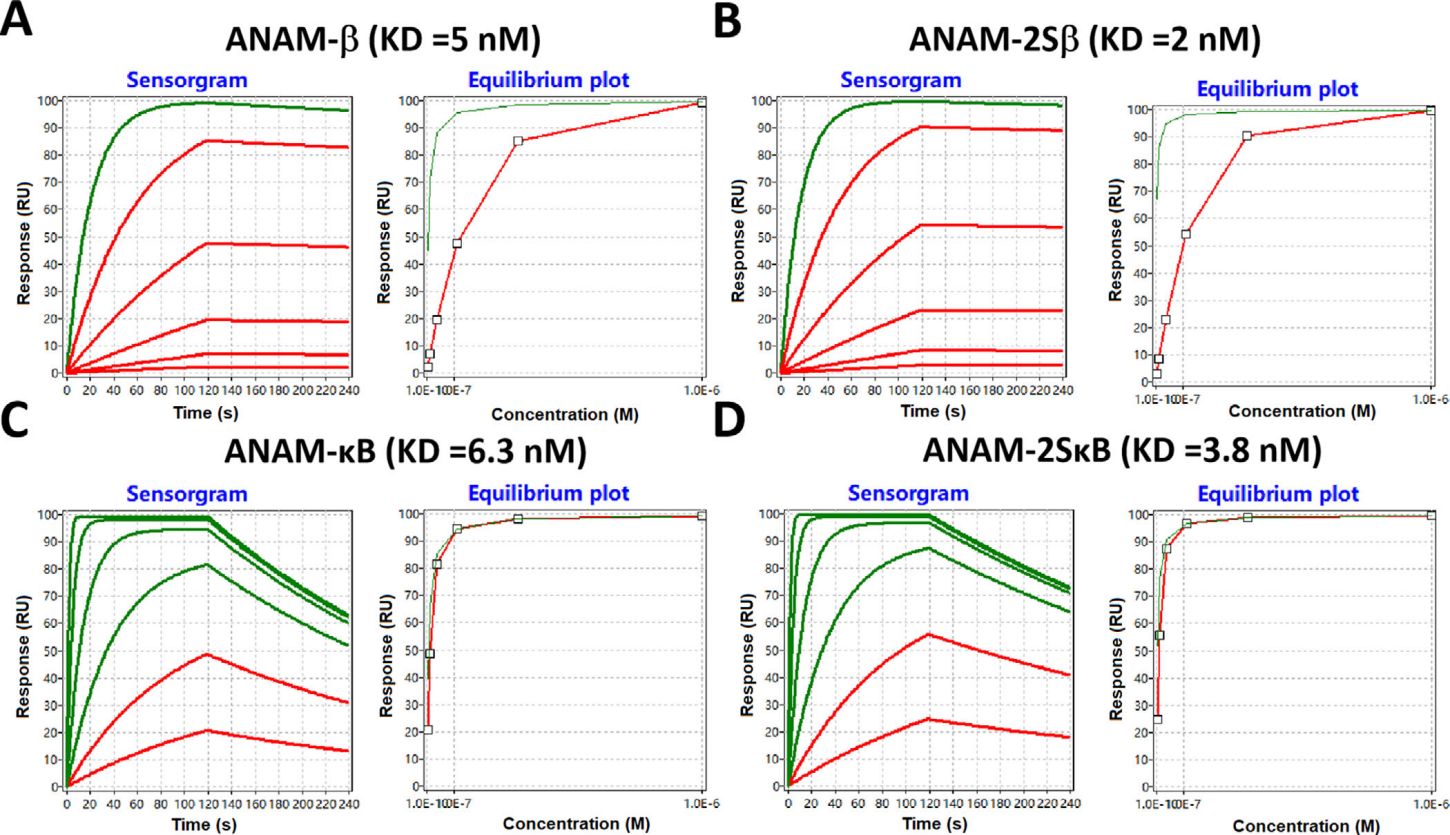
Protein binding characterization of ANAMs. SPR‐based RNA binding characterization was performed. Representative SPR sensorgrams and equilibrium binding plots of the ANAM‐β (A), ANAM‐2Sβ (B), ANAM‐κB (C), and ANAM‐2SκB (D) are shown. The association and dissociation portions from each protein at six different concentrations are included in the sensorgrams. *K*_D_, equilibrium constant. In the sensorgram, the curves that reach less than 95% equilibrium at the end of the analyte injection time are red and the curves above this value are green. The concentrations represented by these curves (from top to bottom) are: 1.0 × 10^−6^ M, 3.3 × 10^−7^ M, 1.1 × 10^−7^ M, 3.7 × 10^−8^ M, 1.2 × 10^−8^ M, and 4.1 × 10^−9^ M, respectively

To further validate the performance of constructed ANAMs, a reporter that sensed β‐catenin transcriptional activity was constructed by specifically subcloning the β‐catenin‐responsive promoter sequences into a dual‐luciferase vector. The results showed that the ratio of firefly luciferase versus Renilla luciferase expression was decreased by ANAM‐β, and that it was more significantly suppressed by ANAM‐2Sβ (Figure S1A). Binding affinities determined by the SPR assay further confirmed that ANAM‐2Sβ had a stronger β‐catenin‐binding ability than ANAM‐β, which provided direct evidence for the mechanism and effect of ANAM (Figure 2A,B).

Next, we constructed several ANAMs, which effectively suppressed the NF‐κB transcription factor. We verified the inhibitory effect of ANAMs on NF‐κB by detecting the expression of its downstream target genes (*TRAF1* and *Bcl‐XL*). We found that when we simply arrayed two or three NF‐κB aptamers and constructed ANAM‐2κB and ANAM‐3κB, neither of them could effectively inhibit NF‐κB (Figure 1E,F). Therefore, to construct an ANAM that produced higher inhibitory effects on NF‐κB when compared to ANAM‐κB, we again inserted the RNA scaffold between the two NF‐κB aptamers to construct ANAM‐2SκB (Figure 1G). As expected, ANAM‐2SκB had a stronger inhibitory effect (about 50% vs 30%) on NF‐κB than ANAM‐κB (Figure 1B,C). To further confirm the activity of constructed ANAMs, a similar dual‐luciferase reporter that sensed NF‐κB transcriptional activity was constructed, and the expression trend of luciferase test results remained the same as that of quantitative PCR (qPCR; Figure S1B). Binding affinities determined by the SPR assay also suggested that ANAM‐2SκB had a stronger NF‐κB‐binding ability than ANAM‐κB (Figure 2C,D).

Our results demonstrated that only arraying multi‐aptamers was ineffective and did not improve the inhibitory effects of the aptamers on the targeted proteins as the gaps between the arrayed multi‐aptamers prevented the formation of a functionally superior structure within cells. The double aptamers stabilized by the scaffold showed a stronger inhibitory effect on target proteins than the single aptamers.

### 2.2 Effects of ANAMs on transgene expression

To investigate whether the expression of the transgene was increased using the ANAMs, we first verified the effect of ANAMs on plasmid expression in 293t and 5637 cells. We used the firefly luciferase protein cassette and enhanced green fluorescent protein cassette (pEGFP), both driven by the relatively weak constitutive HSV‐TK promoter in 293t cells (normal cells) and 5637 cells (cancer cells). A weak promoter was chosen as the tissue/cell‐specific promoters used in many gene therapy cases were often weak promoters, so their gene expression efficiency needed to be further enhanced. Then, we tried to increase the expression efficiency of the reporter gene plasmid using the ANAMs. We introduced point mutations into ANAM‐β and ANAM‐κB at several sites to construct ANAM‐control1 and ANAM‐control2, which could not bind to targets and were set as controls.

**FIGURE 3:**
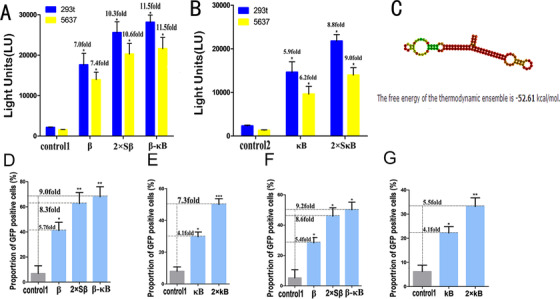
Transgene expression using ANAMs determined by luciferase and GFP reporter genes. A, Effects of ANAM‐β, ANAM‐2Sβ, and ANAM‐β‐κB on improving the efficiency of luciferase transgene expression in 293t and 5637 cells. B, Effects of ANAM‐κB and ANAM‐2SκB on improving the efficiency of luciferase transgene expression in 293t cells and 5637 cells. C, Secondary structure and the nucleic acid sequence of ANAM‐β‐κB. D, Effects of ANAM‐β, ANAM‐2Sβ, and ANAM‐β‐κB on improving the efficiency of GFP transgene expression in 293t cells assessed by flow cytometry. E, Effects of ANAM‐κB, and ANAM‐2SκB on improving the efficiency of GFP transgene expression in 5637 cells assessed by flow cytometry. F, Effects of ANAM‐β, ANAM‐2Sβ, and ANAM‐β‐κB on improving the efficiency of GFP transgene expression in 5637 cells assessed by flow cytometry. G, Effects of ANAM‐κB, and ANAM‐2SκB, on improving the efficiency of GFP transgene expression in 5637 cells assessed by flow cytometry. All experiments were repeated three times. ^*^ *P* < .05, ^**^ *P* < .01, ^***^ *P* < .001 compared to the ANAM control group

We found that ANAM‐β significantly increased the expression level of luciferase compared to the control groups in 293t cells and 5637 cells (Figure 3A). The improved degree of ANAM‐2Sβ in luciferase expression was significantly higher than that of ANAM‐β (Figure 3A). Thus, inhibiting the function of β‐catenin improved the transgene expression of luciferase in 293t cells. Next, we investigated whether luciferase expression efficiency was also improved by inhibiting NF‐κB within cells. We observed that ANAM‐κB increased the expression of luciferase to some extent, whereas ANAM‐2SκB showed a greater ability to increase transgene expression compared with ANAM‐κB (Figure 3B). We also investigated whether the simultaneous inhibition of β‐catenin and NF‐κB could further improve the activation effect on transgene expression. We inserted the scaffold between the aptamer β‐catenin and NF‐κB and constructed the ANAM‐β‐κB (Figure 3C), and found that ANAM‐β‐κB produced a stronger activation effect on relative luciferase expression. ANAM‐β‐κB and ANAM‐2Sβ were similar in their ability to improve the efficiency of transgene expression (Figure 3A), and both constructs showed greater ability than ANAM‐2SκB (Figure 3B). We also tested luciferase expression after transfecting ANAMs with or without β‐catenin/NF‐κB overexpression plasmid. The co‐expression of β‐catenin/NF‐κB led to a significant reduction of luciferase expression in 293t cells with a low expression level of β‐catenin and NF‐κB^31^ (Figure S2).

We also used pGFP expression to test the ability of ANAMs to improve transgene expression in 293t and 5637 cells. The ANAMs (β, κB, 2Sβ, 2SκB, and β‐κB) exhibited varying degrees of ability to increase GFP expression. Relative to the control group, ANAM‐β‐κB showed the greatest ability and ANAM‐β had the weakest ability to improve transgene expression (Figure 3D; Figure S3A); both ANAM‐κB and ANAM‐2SκB also increased pGFP expression. Besides, we found that ANAM‐β‐κB showed significant improvement in pGFP expression compared to group ANAM‐2SκB (Figure 3E; Figure S3B). Increased expression efficiency of the GFP transgene was also observed in 5637 cells. (Figure 3F,G; Figure S3C,D). Therefore, we selected the ANAM‐β‐κB for further experimentation as it exhibited improved transgene expression in both 293t and 5637cells.

To prove that ANAMs are helpful for transgene expression of difficult‐to‐express cellular genes, we constructed an overexpression vector of the CD23 gene that was not expressed in 293t cells. By co‐transfecting it with ANAMs, we found that ANAMs increased the expression efficiency of the CD23 transgene (Figure S4A,B).

These results demonstrated that ANAMs improved the expression of transgenes driven by a weak promoter in both normal and cancer cells. Moreover, we found that the simultaneous inhibition of β‐catenin and NF‐κB signaling pathways showed the greatest improvement of transgene expression.

### 2.3 Effects of ANAMs on type I interferons and inflammatory cytokine expression

To confirm that the enhancement of the transgene effect observed with ANAMs was due to the inhibition of the IIR and not some other off‐target effects, we measured the effects of ANAMs on type I interferons and inflammatory cytokines. IFN‐β, TNF‐α, and IL‐12 were chosen as markers of the IIR activation.^21^, ^26^, ^32^ The 293t and 5637 cell lines were selected to investigate the effect of the cellular IIR system on plasmid DNAs. Theoretically, upon transfection of the cytosolic plasmid DNAs into cells, these IIR markers would increase accordingly. The transfected PcDNA3.1 plasmid vector was used as a template to mimic cytosolic DNAs. That activation of the intracellular IIR system was caused by cytosolic DNAs and not by other translated proteins was confirmed by deleting the extra components (such as the resistance gene and marker protein‐coding gene) in the pcDNA3.1 plasmid vector and constructing a blank pcDNA3.1 vector (bpcDNA3.1).

**FIGURE 4:**
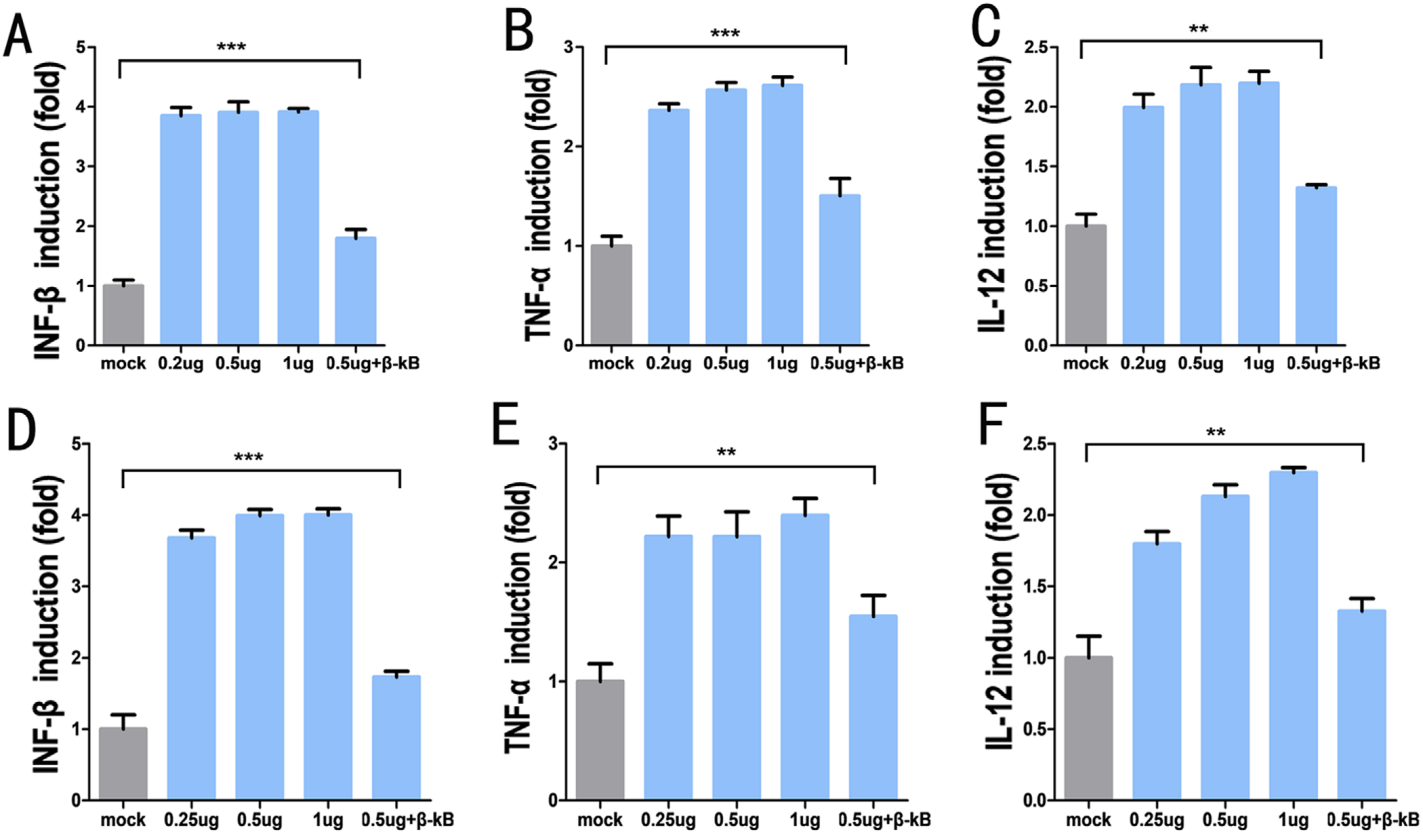
Expression of type I interferons and inflammatory cytokines measured by qPCR. In 293t cells, 0.2 μg, 0.5 μg, 1 μg of plasmid induced the expression of INF‐β (A), TNF‐α (B), and IL‐12 (C). In the presence of ANAM‐β‐κB in 293t cells, 0.5 μg of plasmid slightly induced the expression of INF‐β (A), TNF‐α (B), and IL‐12 (C). In 5637 cells, 0.2 μg, 0.5 μg, 1 μg of plasmid induced the expression of INF‐β (D), TNF‐α(E), and IL‐12 (F). In 293t cells, 0.5 μg of plasmid slightly induced the expression of INF‐β (D), TNF‐α (E), and IL‐12 (F) in the presence of ANAM‐β‐κB. All experiments were repeated three times. ^**^ *P* < .05, ^***^ *P* < .001 compared to the mock group

We found that compared to the mock group, the expression profiles of IFN‐β, TNF‐α, and IL‐12 transfected with bpcDNA3.1 were significantly increased in 293t (Figure 4A‐C) and 5637 cells (Figure 4D‐F). These results confirmed that the existence of cytosolic DNAs activated the IIR system within cells. Next, we used transfection groups with 0.2, 0.5, and 1 μg of bpcDNA3.1 to determine whether the activation of the IIR was dependent on cytoplasmic DNA concentration. We found that the expression levels of IFN‐β, TNF‐α, and IL‐12 in 293t (Figure 4A‐C) and 5637 (Figure 4D‐F) cells were only slightly elevated with increasing concentrations of plasmids with no statistical difference. This observation indicated that a small amount of the transfected plasmids was sufficient to cause significant IIR.

We also investigated the effect of ANAMs on the IIR in cells. We co‐transfected ANAM‐β‐κB and bpcDNA3.1 into 293t (Figure 4A‐C) and 5637 (Figure 4D‐F) cells and measured the expression levels of IFN‐β, TNF‐α, and IL‐12. The results showed that ANAM‐β‐κB had a significant inhibitory effect on IFN‐β, TNF‐α, and IL‐12. Moreover, the data also suggested that the expression profiles of IFN‐β, TNF‐α, and IL‐12 in the β‐κB group were not significantly different from those in the mock group in which the cells were not transfected with any plasmid DNAs. These data showed that the presence of cytosolic DNA activated the intracellular IIR system and limited the expression of plasmids in the cells. Also, ANAM‐β‐κB had significant inhibitory effects on the markers of the IIR system (IFN‐β, TNF‐α, and IL‐12), and suppressed their expression to the background level in the mock group.

### 2.4 Effects of ANAMs on improving the performance of the CRISPR‐Cas9 system

**FIGURE 5:**
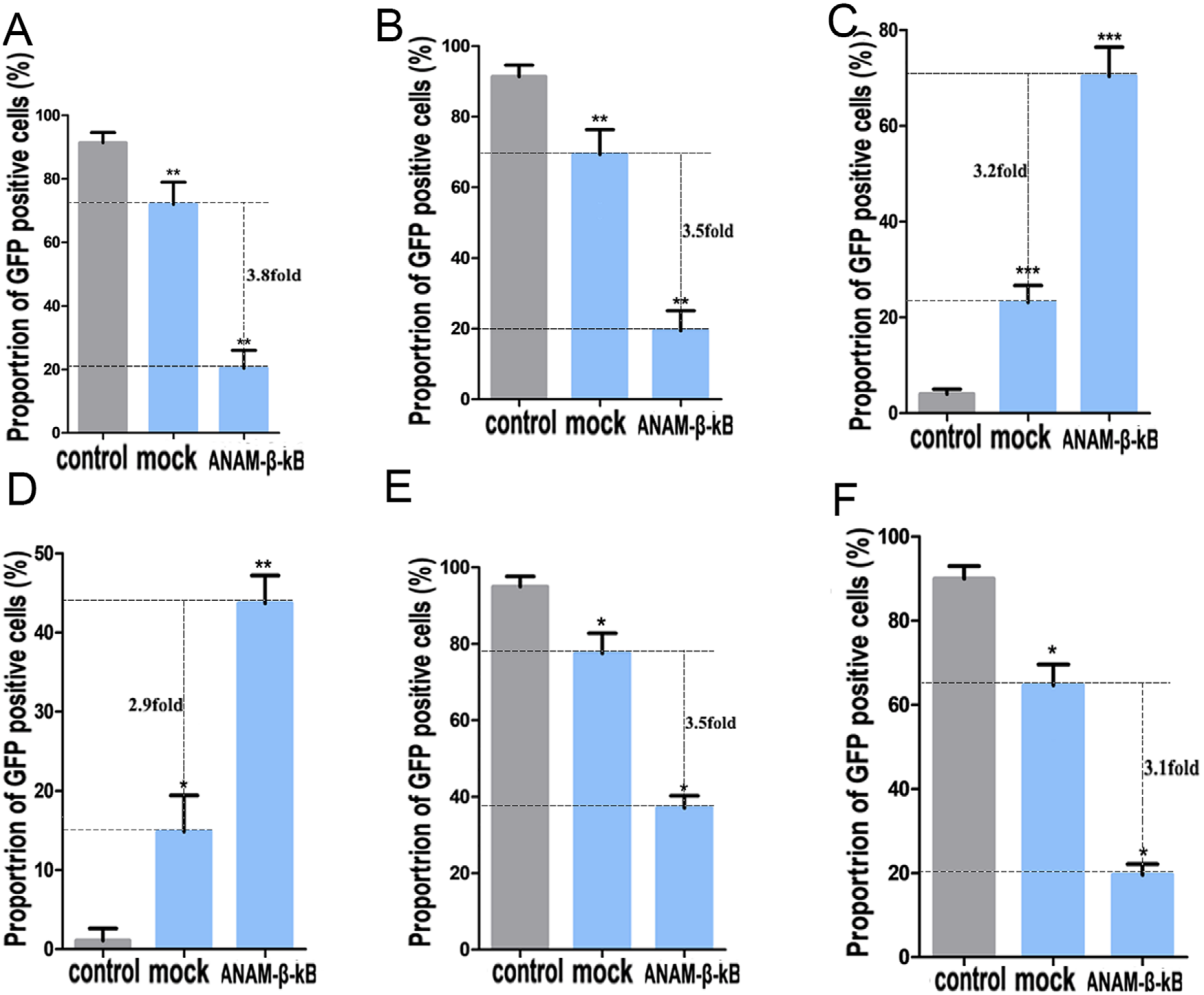
Gene editing efficiency of CRISPR‐Cas9 and its derivative system determined by the GFP reporter gene. Improved efficiency of CRISPR‐Cas9 gene editing in 293tand 5637 cells by ANAM‐β‐κB. Flow cytometry was used to determine the effects of ANAM‐β‐κB in 293t cells (A) and 5637 cells (B). Improved efficiency of CRISPR‐dCas9‐vp64 activation in 293t and 5637 cells by ANAM‐β‐κB. Flow cytometry was used to determine the effects of ANAM‐β‐κB in 293t cells (C) and 5637 cells (D). Improved efficiency of CRISPR‐dCas9‐Krab repression in 293t and 5637 cells by ANAM‐β‐κB. Flow cytometry was used to determine the effects of ANAM‐β‐κB in 293t cells (E) and 5637 cells (F). All experiments were repeated three times. ^*^ *P* < .05, ^**^ *P* < .01, ^***^ *P* < .001 compared to the ANAM control group

The discovery and development of the CRISPR‐Cas9 system have revolutionized gene editing, and its widespread use has enabled making phenotypic changes in eukaryote cells at the genetic level.^5^, ^33^ Each element of the CRISPR‐Cas9 system is transported into the cells in a plasmid vector for gene editing. In this study, we attempted to improve the gene‐editing efficiency of the CRISPR‐Cas9 system by using ANAMs to improve its knockdown effect on targeted DNAs. The CRISPR‐Cas9 system‐mediated DNA knockout by producing DNA breaks, which induced non‐homologous end‐joining (NHEJ), resulting in the ineffective expression of targeted genes. We set GFP as the targeted gene and used the plasmid vector to transport the CRISPR‐Cas9 system to act on it. The ANAMs were used to improve CRISPR‐Cas9 transgene expression and the gene‐editing efficiency of the system in cells. The stably transfected cell lines, 293t‐GFP and 5637‐GFP, which were capable of continuously expressing GFP, were generated and transfected with the plasmid (pCRISPR‐Cas9) carrying the CRISPR‐Cas9 system. We detected a significantly higher degree of GFP inactivation in the ANAM‐β‐κB group than in the other control groups (Figure S5A,B). At the same time, we evaluated the expression level of GFP in cells by flow cytometry (Figure 5A,B). The CRISPR‐Cas9 system specifically caused cleavage of the target gene of interest and caused NHEJ, which resulted in the deletion or insertion of nucleotide pairs in the targeted gene, forming the frameshift mutations, and achieving gene knockout.^34^ The data suggested that the β‐κB group had a significantly higher knockdown percentage than the other groups, which indicated that ANAM‐β‐κB significantly improved the gene‐editing efficiency of CRISPR‐Cas9 system.

To further determine whether ANAMs could also increase homology‐directed repair (HDR) mediated by CRISPR‐Cas9, an EGFP reporter reconstitution assay was used as previously described.^35^ The mutant EGFP (379A>T, 384C>G) reporter gene containing a premature stop codon was integrated into the genome of 293t cells. EGFP expression could be restored when the mutation was repaired by HDR using a donor sequence, including the wild type EGFP template. The 293t mutant EGFP cells were co‐transfected with sgRNA‐mutant EGFP/Cas9 and ANAM‐β‐κB. The results showed that ANAM‐β‐κB significantly improved the homologous recombination mediated by the CRISPR‐Cas9 system (Figure S6).

We selected two previously reported target genes^35^ (DNMT1 and MED7) to explore whether ANAMs could increase CRISPR knock‐out in stem cells known for low efficiencies^36^ and tested the genome‐editing capacity in human induced pluripotent stem (iPS) cells. Based on PCR and TIDER analyses, we observed the highest indel rates (35.3% for DNMT1 and 31.6% for MED7) in the ANAM‐β‐κB group, whereas in the mock group without ANAM, indel rates for DNMT1 and MED7 were only 15‐20% (Figure S7A,B).

A key feature of Cas9 is its ability to bind to DNA at a site defined by both guide RNA and the PAM, enabling permanent modification beyond fixed‐point cleavage of the targeted DNA.^37^ Specifically, the catalytically deactivated version of Cas9 (dCas9) has been widely used for the regulation of targeted genes throughout the genome.^38^ Although dCas9 can not induce DNA cleavage, it remains the ability to bind to DNA sequence and thereby regulates gene transcription by fusing different transcriptional regulators. We previously reported dCas9‐vp64 (transcriptional activator) and dCas9‐KRAB (transcriptional repressor) fusion proteins and used them to activate and suppress the GFP signal, respectively.^39^ In the present study, we constructed an all‐or‐nothing promoter (AON promoter) to drive the expression of the downstream gene of interest (GFP or luciferase), and used dCas9‐vp64 to induce this expression system (Table S3 and Figure S8). The well developed dCas9‐KRAB was used to target the sequence of the GFP upstream promoter and suppress the expression of GFP in 293t‐GFP and 5637‐GFP. We generated stably transfected 293t and 5637 cell lines, which continuously expressed AON promoter‐related expression system (293t‐AON and 5637‐AON). Next, we transfected the CRISPR‐dCas9‐VP64 or CRISPR‐dCas9‐KRAB plasmid into the cell lines carrying a particular expression system. The change in fluorescence intensity of GFP was monitored to evaluate whether ANAMs affected the transcriptional regulatory efficiency of CRISPR‐dCas9. Furthermore, we measured the fluorescence intensity of GFP by flow cytometry sorting. In the AON promoter‐related expression system, under the control of CRISPR‐dCas9‐VP64, the AON promoter successfully activated and drove the expression of GFP. Also, there was a significant improvement of GFP expression in the β‐κB group relative to the other control groups (Figure 5C,D; Figure S5C,D). In the dCas9‐KRAB suppression system, we also found that, under the action of ANAM‐β‐κB, GFP had a weak expression level, which also meant that CRISPR‐dCas9‐KRAB had a significant inhibitory effect on the β‐κB group (Figure 5E,F; Figure S5E,F).

These data suggested that ANAM improved the gene‐editing efficiency of both CRISPR‐Cas9 and CRISPR‐dCas9. As we had shown in the previous section, ANAM might improve the expression of the CRISPR‐Cas9 transgene by inhibiting the IIR system of cells, thereby improving the gene‐editing efficiency.

### 2.5 Practical applications of ANAMs on CRISPR‐Cas9 for improving gene editing in cancer cells

**FIGURE 6:**
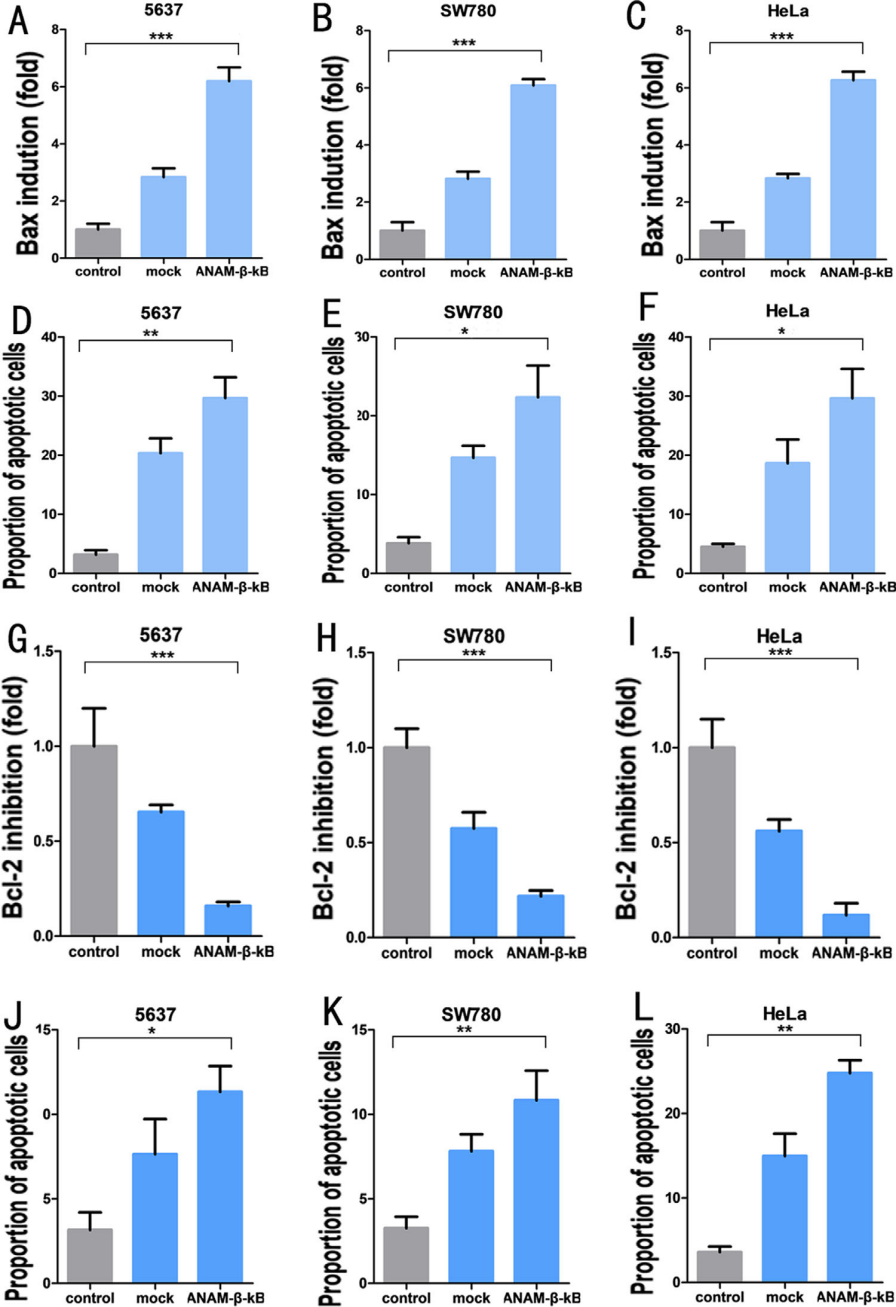
Application of ANAM‐β‐κB in different types of cancer cells. qPCR assay showing that CRISPR‐dCas9‐vp64 activated the expression of the *Bax* gene in 5637 cells (A), SW780 cells (B), and HeLa cells (C) (mock groups). ANAM‐β‐κB improved the activation efficiency of the *Bax* gene by CRISPR‐dCas9‐vp64 in 5637 cells (A), SW780 cells (B), and HeLa cells (C) (ANAM‐β‐κB groups). Flow cytometry suggesting that the proportion of corresponding apoptotic cells varied with the expression of the *Bax* gene in 5637cells (D), SW780 cells (E), and HeLa cells (F). qPCR assay showing that CRISPR‐dCas9‐KRAB inhibited the expression of the *Bcl‐2* gene in 5637 cells (G), SW780 cells (H), and HeLa cells (I) (mock groups). ANAM‐β‐κB improved the inhibitory efficiency of the Bcl‐2 gene by CRISPR‐dCas9‐KRAB in 5637 cells (G), SW780 cells (H), and HeLa cells (I) (ANAM‐β‐κB). Flow cytometry analysis suggested that the percentage of apoptotic cells varied with the expression of the *Bcl‐2* gene in 5637cells (J), SW780 cells (K), and HeLa cells (L). All experiments were repeated three times. ^*^ *P* < .05, ^**^ *P* < .01, ^***^ *P* < .001

To investigate the practical applications of ANAMs on CRISPR‐Cas9, we used ANAMs in vitro to determine whether they improved the gene editing of CRISPR‐Cas9 on target genes of interest in cancer cells. Bax protein serves as a hallmark of cell apoptosis and is inhibited by overexpressing Bcl‐2 protein in cancer cells, contributing to tumor development and inhibition of cell apoptosis.^40^ We used the CRISPR‐Cas9 system to interfere with Bcl‐2/Bax in cancer cells to promote cell apoptosis. Furthermore, we sought to improve the gene‐editing efficiency of CRISPR‐Cas9 in 5637, SW70, and Hela cells using ANAMs. We analyzed the upstream promoter sequence of the *Bax* gene and designed a sgRNA specific to this gene. The well‐developed CRISPR‐dCas9‐VP64 system was used to target the upstream promoter of the *Bax* gene to overexpress the Bax protein and induce cell apoptosis in cancer cells. After transfecting the CRISPR‐dCas9‐VP64 system into cancer cells for 48 h, we measured the mRNA level of the *Bax* gene and analyzed cell apoptosis by flow cytometry. The CRISPR‐dCas9‐VP64 system successfully induced the expression of the *Bax* gene in cancer cells (Figure 6A‐C), and the overexpressed *Bax* protein led to increased cell apoptosis (Figure 6D‐F; Figure 9A‐C).

Next, we tested the gene‐editing efficiency of CRISPR‐dCas9‐VP64 using ANAMs, which exerted a greater activation effect on the *Bax* gene. In the presence of ANAMs, the mRNA (Figure 6A‐C) and protein levels of *Bax* (Figure S10A‐C) were increased. We also used flow cytometry to analyze the effect of the Bax protein on apoptosis and observed that ANAMs increased the activation effect of CRISPR‐dCas9‐VP64 on the *Bax* gene and promoted cell apoptosis (Figure 6D‐F; Figure S9A‐C).

We also investigated the application of ANAMs to improve the gene‐editing efficiency of CRISPR‐Cas9 in cancer cells. The CRISPR‐dCas9 system was used to downregulate the expression of the *Bcl2* gene and to promote *Bax* gene expression, which induced apoptosis of tumor cells. The expression of the *Bcl2* after transfection of CRISPR‐dCas9‐KRAB was measured (Figure 6G‐I), and the effect of down‐regulation of the *Bcl2* gene on cancer cells was analyzed by flow cytometry (Figure 6J‐L; Figure S9D‐F). We used ANAMs to increase the efficiency of CRISPR‐dCas9‐KRAB and observed greater transcriptional inhibition efficiency, which resulted in lower expression of the *Bcl2* gene. The protein level of *Bcl2* was also decreased by ANAMs (Figure S10D‐F). Similarly, flow cytometry results showed that apoptosis in cancer cells increased with the decrease in *Bcl2* expression.

Together, these results indicated that ANAMs were equally effective in various cancer cells, and improved the gene‐editing efficiency of CRISPR‐Cas9.

## 3 DISCUSSION

With the widespread application of CRISPR‐Cas9, a range of methods has been developed to improve the gene‐editing efficiency. More powerful delivery vectors have been developed to increase the quantity of the CRISPR‐Cas9 systems within cells. For example, the artificial adeno‐associated virus and lipid nanoparticles were designed as novel and efficient vectors, which could more efficiently transport the CRISPR‐Cas9 system into cells.^41^, ^42^, ^43^ The CRISPR‐Cas9 system's effect on knocking out the target gene was improved by optimizing the structure of the single guide RNA (sgRNA) of CRISPR‐Cas9.^44^, ^45^ These methods have enhanced the gene‐editing efficiency of CRISPR‐Cas9 to some extent.

In many studies aimed at improving the gene editing‐efficiency of CRISPR‐Cas9, the focus has been on the transcription level with little attention to the potential regulation at the posttranscriptional level. In the present study, we determined the gene‐editing ability of different previously characterized CRISPR‐Cas9 systems in vitro using ANAMs, which involved inhibition of the IIR system within mammalian cells. We used ANAMs in cancer cells to improve the gene‐editing activity of CRISPR‐Cas9 for specific genes. Our results showed that ANAMs improved the gene‐editing activities of the CRISPR‐Cas9 system and its derivatives for almost any target.

It is interesting that arraying multiple aptamers with a simple connection not only failed to achieve an additive effect but also caused a lack of functionality. One possible explanation for this phenomenon would be that in arrayed multiple aptamer clusters, the original aptamer sequence failed to fold into an effective construction. This observation was consistent with an earlier study, which suggested that different RNA modules interacted with each other, causing the RNA module to function less efficiently.^46^ We, therefore, designed an RNA scaffold and inserted it between two aptamers, which stabilized the structure of arrayed multiple aptamer hairpins. We used the GFP and luciferase expression cassettes to test the effect of ANAMs on transgene expression. The results demonstrated increased expression levels of both GFP and luciferase were achieved under the influence of ANAMs. These data also showed that ANAMs not only improved the gene‐editing efficiency of CRISPR‐Cas9 but also improved the transgene expression efficiency associated with plasmid transfection. This conceptually simple idea could be utilized in a biological kit that facilitates the construction of RNA molecules to simultaneously inhibit different signaling proteins and to develop strategies for transgene therapy of diseases. Thus, ANAMs could be utilized for the formation of RNA modules that specifically inhibit the desired signaling proteins. In addition, ANAMs could also be used to reveal the biological function of a signaling pathway or to study RNA‐protein interactions. The only limitation is that there were not many aptamers available to bind to cellular proteins. Perhaps in future works, we could consider using protein binding elements contained in long noncoding RNAs, which would be a good way to solve this problem.

Although this research cannot be directly applied to the diagnosis and treatment of diseases, it is possible to consider the methods involved in our work when applying CRISPR‐Cas9 technology in vivo. The application potentials of CRISPR‐(d)Cas9 in gene therapy is a fascinating area of research, but the in vivo CRISPR efficacy represents one of the major drawbacks.^47^, ^48^ Artificial nucleic acid molecules can be co‐expressed with CRISPR‐Cas9 on the same vector, thereby improving the efficiency of the CRISPR technology. The biosafety of the selected delivery system should also be considered when applied to in vivo studies. Several studies have described aptamer‐based strategies in CRISPR‐based genome editing and regulation.^49^, ^50^ Therefore, it is possible to combine the aptamer‐modified guide RNA strategy with ANAMs, which might synergistically improve gene editing. Future works are still needed to test the efficacy of ANAMs at the staving of the innate immune response and enhancing CRISPR activity in live tissues.

In summary, we have shown, for the first time, that systematic inhibition of the IIR system within mammalian cells resulted in significant improvements in transgene expression, increasing the expression of CRISPR‐Cas9 plasmids and improving the gene‐editing efficiency. Furthermore, we developed a simple molecular scaffold that stabilized the construction of multi‐hairpin aptamers within cells. Using this approach, we developed several ANAMs that inhibited target molecules at the posttranslational level. Our approach provides an easy strategy for increasing the expression of transgenes, which should improve the effectiveness of clinical gene therapy.

## 4 MATERIALS AND METHODS

### 4.1 Cell culture and cell transfection

HEK293T, 5637, SW80, and Hela cell lines were purchased from American Type Culture Collection (ATCC) (Manassas, VA, USA). HEK293T, SW780, and Hela cells were maintained in Dulbecco's Modified Essential Medium, while 5637 cells were maintained in 1640 medium. All media were supplemented with 10% fetal bovine serum (Invitrogen, Carlsbad, CA, USA) and maintained in 5% CO_2_ at 37°C. For transient transfection assays, the cells were seeded in six‐well plates the day before transfection using Lipofectamine‐3000 (Invitrogen). At 48 h post‐transfection, cells were harvested for follow‐up assays.

The human iPS cell line was purchased from ATCC and cultured in mTeSR1 (STEMCELL Technologies) in Geltrex (Gibco)‐coated six‐well plates. Cells were transfected with plasmids using Lipofectamine Stem Reagent (Invitrogen) and were harvested for follow‐up assays at 48 h post‐transfection.

### 4.2 Plasmid construction

All ANAMs were constructed by chemical synthesis based on the designed sequence. The ANAM sequences are listed in Table S1. The U6 promoter was used to drive the expression of ANAMs, and the U6‐ANAMs were incorporated into a pcDNA3.1 plasmid vector. The original plasmids (Addgene) of CRISPR‐Cas9, CRISPR‐dCas9‐vp64, and CRISPR‐dCas9‐Krab used in this study were previously reported.^39^ The bpcDNA3.1 sequence was designed based on the sequence of pcDNA3.1 after deleting the resistance gene and marker protein‐coding gene from its backbone and was then chemically synthesized. β‐Catenin‐pcDNA3.1, NF‐κB‐pcDNA3.1, and CD23‐pcDNA3.1 plasmids were purchased from Beijing SyngenTech Co., Ltd.

### 4.3 Generation of the stable transgenic cell lines

The 293t and 5637 cell lines were transfected with pcDNA3.1/GFP/Neo plasmids using Lipofectamine 3000 (Invitrogen) and treated with G418 to select the 293t‐GFP and 5637‐GFP stable transgenic cell lines. Also, the 293t and 5637 cell lines were transfected with the AON promoter‐GFP expression pcDNA3.1 plasmid to generate the 293t‐AON and 5637‐AON stable transgenic cell lines. The stable transgenic cell lines were obtained by G418 resistance screening.

### 4.4 RNA extraction and real‐time qPCR

The total RNAs of transfected cells were extracted using TRIzol reagent (Invitrogen) according to the manufacturer's protocol. The RevertAid First Strand cDNA Synthesis Kit (Fermentas, Hanover, MD, USA) was used to synthesize cDNAs from total RNAs. The All‐in‐One qPCR Mix (GeneCopoiea, Rockville, MD, USA) was used to perform the real‐time qPCR reactions on an ABI PRISM 7000 Fluorescent Quantitative PCR System (Applied Biosystems, Foster City, CA, USA). The PCR cycling parameters were as follows: 95°C for 15 min, followed by 40 cycles of 95°C for 15 s, 60°C for 30 s, and 72°C for 45 s. The primer sequences are listed in Table S2.

### 4.5 Dual‐luciferase reporter assay

To measure the transcriptional activity of β‐catenin and NF‐κB, a dual‐luciferase reporter was constructed using β‐catenin or NF‐κB responsive elements. The β‐catenin reporter contained Tcf‐binding elements and the minimal promoter, which were inserted upstream of the firefly luciferase gene on the dual‐reporter vector. Similarly, the NF‐κB reporter contained multiple binding sites for NF‐κB and the minimal promoter. Cells were seeded in six‐well plates (5 × 10^5^ per well) and co‐transfected with the β‐catenin or NF‐κB dual‐luciferase reporter vector and the ANAM expression vector. Luciferase activity was measured using the dual‐luciferase assay system (Promega, Madison, WI, USA) according to the manufacturer's instructions at 48 h after transfection. The firefly luciferase activity was normalized to the Renilla luciferase activity.

### 4.6 Surface plasmon resonance assay

The affinity of the constructed ANAMs for β‐catenin or NF‐κB proteins was measured by surface plasmon resonance using a Biacore X100 instrument (GE Healthcare, Uppsala, Sweden). Briefly, the CM5 sensor chip (GE Healthcare) was preequilibrated with running buffer prior to the immobilization of ligands onto the chip surface and activated with 0.05 M *N*‐hydroxysuccinimide (NHS; GE Healthcare) and 0.2 M 1‐ethyl‐3‐(3‐dimethylaminopropyl) carbodiimide (EDC; GE Healthcare). Each protein was then injected into the flow cells of the sensor chip, and amine coupling chemistry was used for covalently attaching ligands to the sensor chip surface. After immobilization of the ligand, the chip surface was deactivated with 1 M ethanolamine hydrochloride to block the remaining unreacted groups. DNA template oligonucleotides for ANAMs were transcribed into RNA using the MEGA‐shortscript T7 kit (Life Technologies). Then various concentrations of the ANAMs were injected over the sensor surface for 1.5 min at 5 μL/min, and subsequently analyzed for equilibrium binding properties.

### 4.7 ELISA assay

The cells were seeded in 6‐well plates (5 × 10^5^ cells/well) and transfected using the firefly luciferase reporter system. After 36 h, the medium was removed, and the cells were lysed with lysis buffer (Analytical Luminescence Laboratories, Mansfield, MA, USA). The level of firefly luciferase activity was measured by the Firefly Luciferase Reporter Assay System (Promega, Madison, WI, USA) according to the manufacturer's protocol.

### 4.8 Western blotting

Cells were washed with phosphate‐buffered saline (PBS) and lysed in the RIPA buffer (Beijing Solarbio Science and Technology Co.). The protein concentration was determined using a BCA kit (Beijing Solarbio Science and Technology Co., Ltd.). Equal amounts of whole protein extracts were electrophoresed onto SDS–polyacrylamide gels and then transferred to polyvinylidene fluoride membranes (Millipore, Billerica, MA). The samples were blocked in 5% dry milk and incubated overnight with the primary antibodies against BCL2 (ab32124; 1:1000) and BAX (ab32503; 1:10 000). The next day, samples were incubated with the horseradish peroxidase‐conjugated secondary antibody (Amersham, Piscataway, NJ) and immunoblots were developed with Super Signal chemiluminescence reagents (Pierce Chemical Co.).

### 4.9 Flow cytometry

To determine fluorescent protein expression, the cells were suspended in PBS and analyzed by flow cytometry (BD Biosciences, San Jose, CA, USA). We also used flow cytometry to determine the level of apoptosis. The cells were resuspended and treated with fluorescein isothiocyanate (FITC) and propidium iodide (PI) dye (Transgene, Beijing, China) according to the manufacturer's instructions. Data were analyzed using FlowJo software (TreeStar, Ashland, OR, USA).

### 4.10 Detection of GFP expression

The transfected cells were cultured in the normal growth medium, and the GFP expression was visualized using fluorescent microscopy (MicroPublisher 3.3 RTV; Olympus, Tokyo, Japan). The images were captured using the auto‐exposure mode.

### 4.11 Determination of NHEJ‐mediated indel mutations

Human iPS cells were harvested at 48 h post‐transfection, and the genomic DNA was extracted using the QuickExtract DNA Extraction system (Epicentre). Subsequently, PCR was performed to amplify the target regions using the genomic DNA as a template. The PCR products were purified using the ISOLATE II PCR and Gel Kit (Bioline) and subjected to Sanger sequencing. Total NHEJ frequencies were determined by decomposition of the sequencing chromatogram using the TIDE software program (https://tide-calculator.nki.nl/). Depicted values were generated from TIDER analyses with *R* ^2^ values > 0.9 and *P* < .001.

### 4.12 Statistical analyses

Data were summarized as the mean ± SEM. Significance tests were performed using SPSS statistical software for windows, version 21.0 (SPSS, Chicago, IL, USA). Statistical significance was determined using Student's *t*‐test or analysis of variance and a value of *P* < .05 was considered to be statistically significant.

## AUTHOR CONTRIBUTIONS

H.Z. and A.L. performed experiments and data analysis. W.H. and Z.C. provided experimental materials and platforms. Y.L. designed and supervised the project and wrote the paper. Y.L. provided financial support for the project.

## CONFLICT OF INTEREST

All authors declare that there is no conflict of interest.

## Supporting information

Supporting information

## Data Availability

The data were available upon reasonable request.
