# CORRIGENDUM

**DOI:** 10.1002/ctm2.344

**Published:** 2021-03-04

Two authors, Mengting Ding and Qun Zhou were added to Zhan et al.^1^

The author byline would thus read:

Hengji Zhan^1,2,†^, Mengting Ding^1,2,†^, Qun Zhou^1,2,†^, Aolin Li^1,2,†^, Zhiming Cai^1,2^, Weiren Huang^1,2^,Yuchen Liu^1,2^

In addition, the following should be added as a footnote:

^†^Hengji Zhan, Mengting Ding, Qun Zhou, and Aolin Li contributed equally to this work

The online version of this article was corrected.

The authors regret these errors.
