# Supplementary material for: Improving transgene expression and CRISPR‐Cas9 efficiency with molecular engineering‐based molecules
Source: Clin Transl Med. 2020 Oct 4;10(6):e194. doi: 10.1002/ctm2.194 (PMC7533053; doi:10.1002/ctm2.194)
Supplement: Supplementary file 1 — Supporting information [file CTM2-10-e194-s001.docx]

**
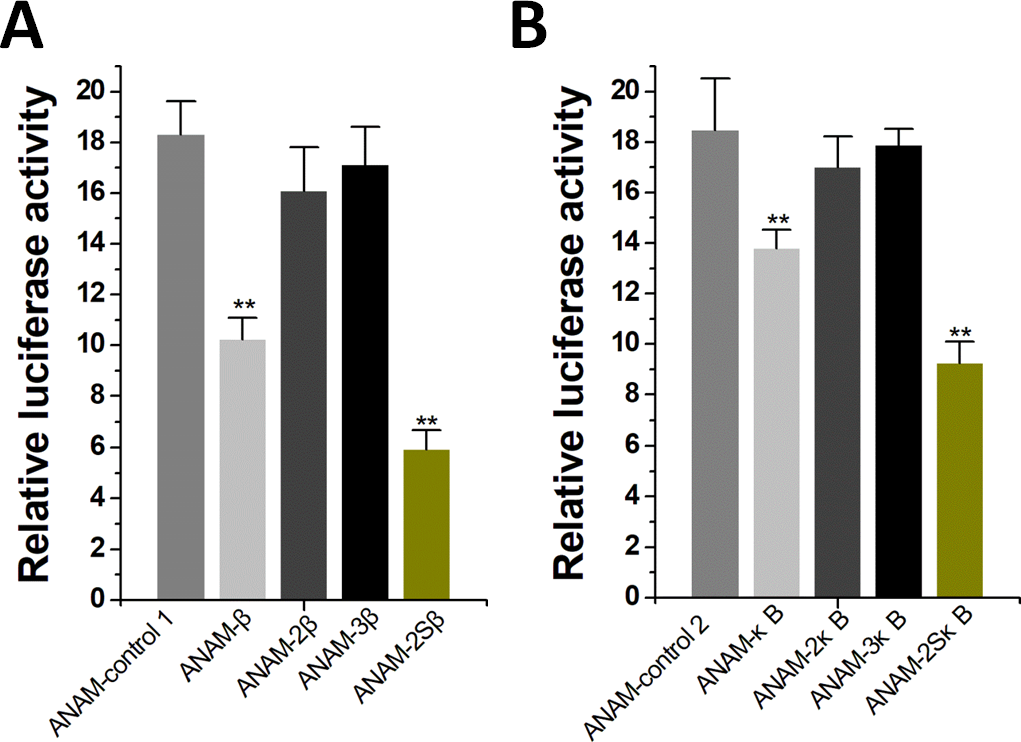
**

**Supplementary Figure 1**.

**Repression of dual-luciferase reporters by ANAMs in 5637 cells**. Luciferase expression levels of 5637 cells co-transfected with the β-catenin (A) or NF-κB (B) reporter and the ANAM expression vector. The firefly luciferase activity was normalized to the Renilla luciferase activity (firefly luciferase/Renilla luciferase) and presented as relative luciferase activity. Results are shown as mean ± SD. ** P < 0.01, compared with ANAM control.





**Supplementary Figure 2**.

**The luciferase expression was determined after coexpression of ANAMs and β-catenin /NF-κB.** The co-expression of β-catenin /NF-κB led to a significant reduction of luciferase expression in 293t cells. Results are shown as mean ± SD. All experiments were repeated three times. (**p<0.01, compared to the ANAM control group).

**
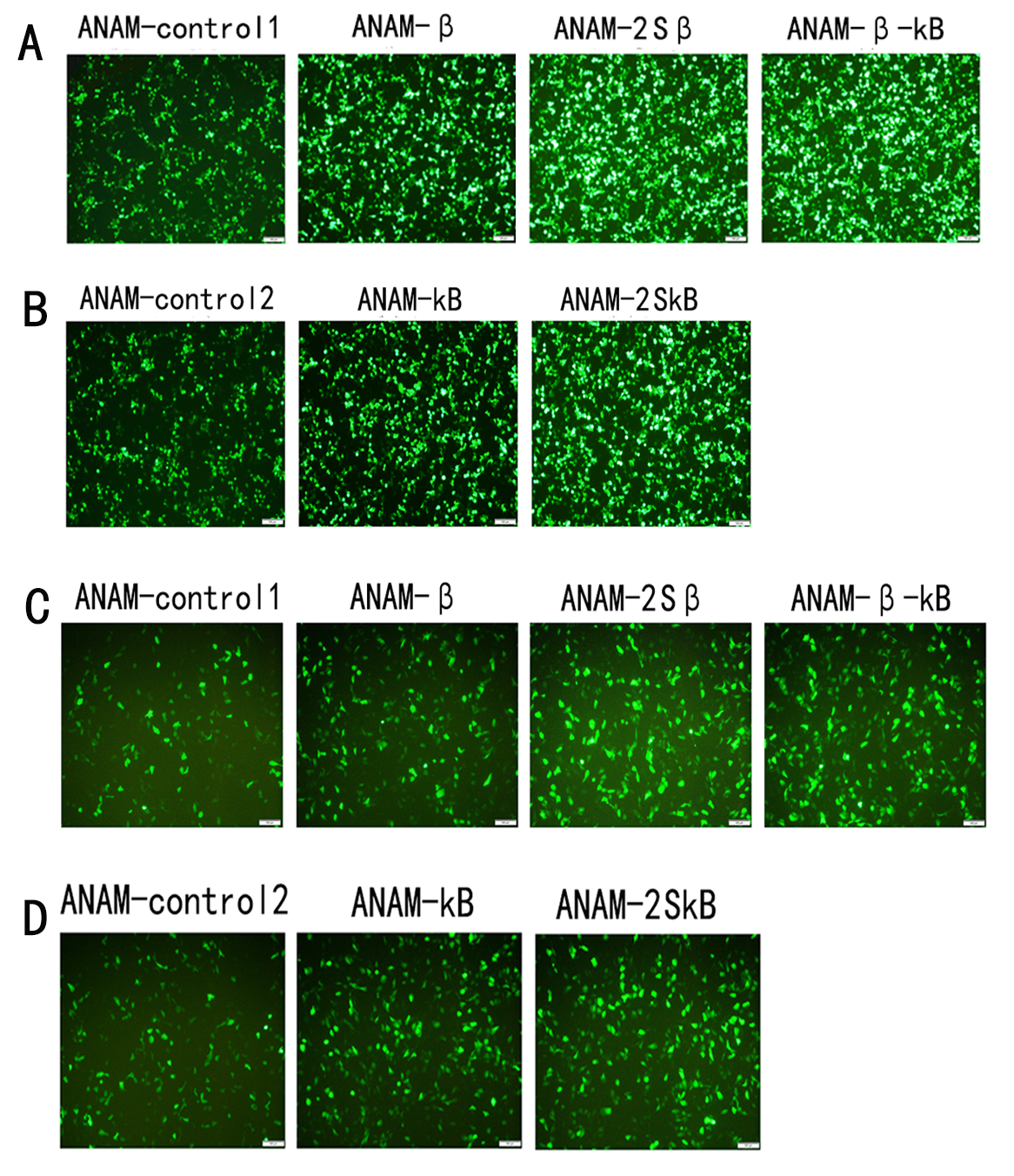
**

**Supplementary Figure 3**.

**The fluorescence microscopy of GFP reporter genes.** (A) The effects of ANAM-β, ANAM-2Sβ and ANAM-β-κB on improving the efficiency of GFP transgene expression in 293t cells, as assessed by fluorescence microscopy. (B) The effects of ANAM-κB, and ANAM-2SκB on improving the efficiency of GFP transgene expression in 293t cells, as assessed by fluorescence microscopy. (C) The effects of ANAM-β, ANAM-2Sβ and ANAM-β-κB on improving the efficiency of the GFP transgene expression in 5637 cells, as assessed by fluorescent microscopy. (D) The effects of ANAM-κB, and ANAM-2SκB on improving the efficiency of GFP transgene expression in 293t cells, as assessed by fluorescence microscopy.


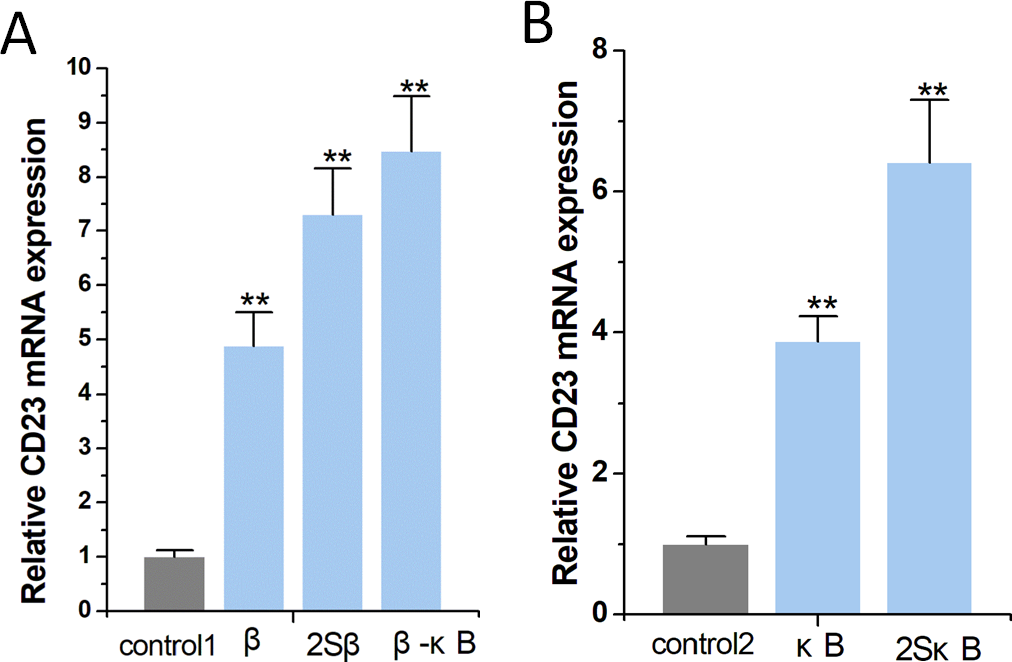


**Supplementary Figure 4**

**The effects of ANAMs on CD23 transgene expression.** (A) The effects of ANAM-β, ANAM-2Sβ, and ANAM-β-κB on improving the efficiency of CD23 transgene expression in 293t cells. (B) The effects of ANAM-κB and ANAM-2SκB on improving the efficiency of CD23 transgene expression in 293t cells. Results are shown as mean ± SD. All experiments were repeated three times. (**p<0.01, compared to the ANAM control group).

**
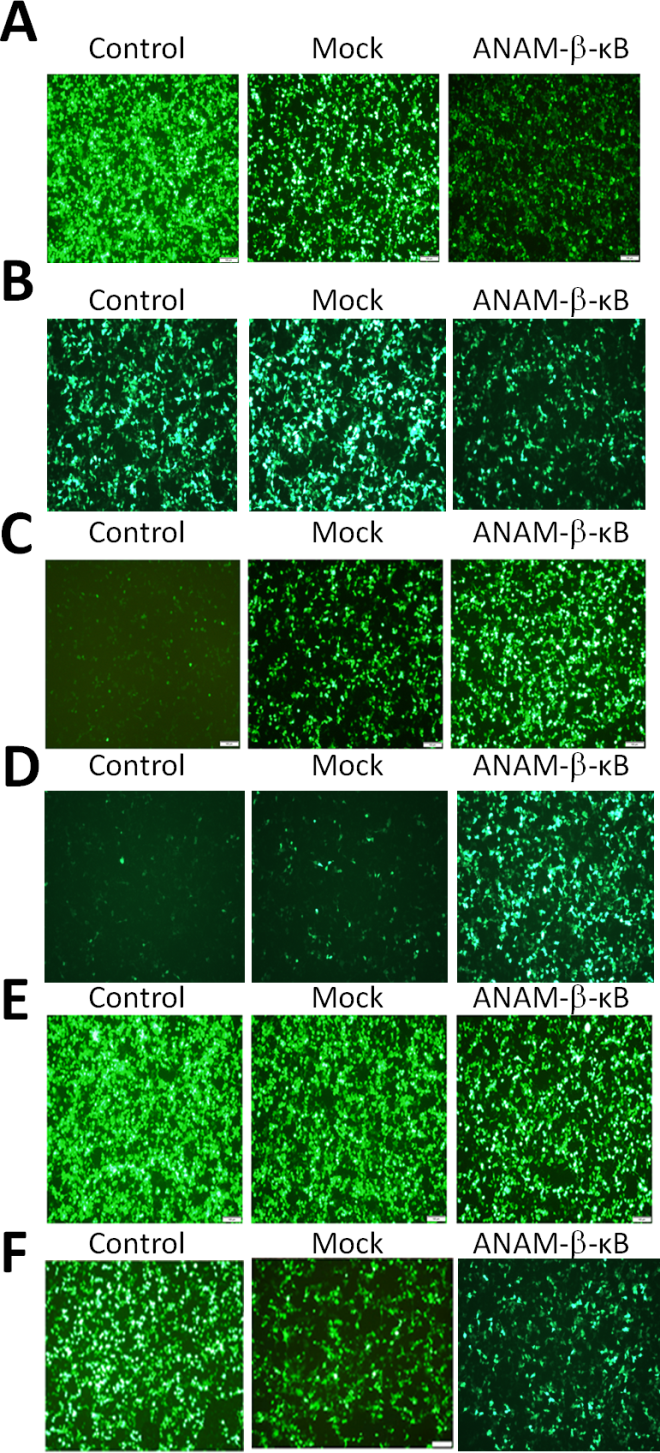
**

**Supplementary Figure 5**

**The fluorescence microscopy of GFP reporter genes.** The effects of ANAM-β-κB on improving the efficiency of the CRISPR-Cas9 gene-editing in 293t cells (A) and 5637 cells (B), as assessed by fluorescence microscopy. The effects of ANAM-β-κB on improving the efficiency of CRISPR-dCas9-vp64 activation in 293t cells (C) and 5637 cells (D), as assessed by fluorescence microscopy. The effects of ANAM-β-κB on improving the efficiency of CRISPR-dCas9-Krab repression in 293t cells (E) and 5637 cells (F), as assessed by fluorescence microscopy.


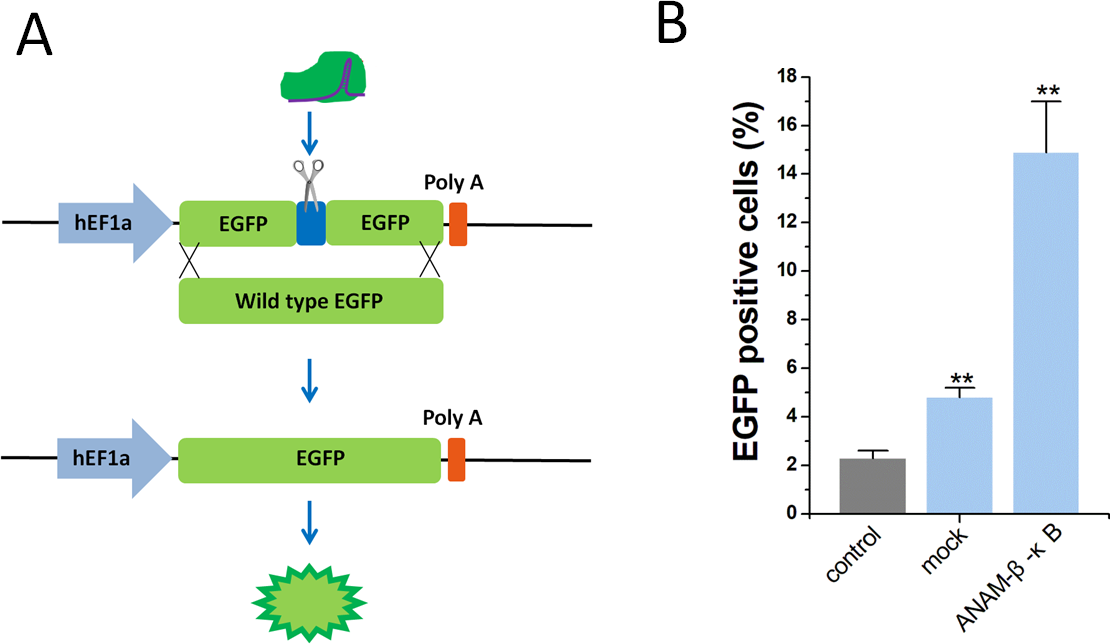


**Supplementary Figure 6**

**Detection of the effect of ANAM on the efficiency of homology-directed repair mediated by CRISPR-Cas9.** (A) Diagram of reconstitution of EGFP domains for detection of homology-directed repair. Homologous recombination between the introduced wild type EGFP donor and the cleaved mutant EGFP target results in the reconstitution of a full-length EGFP gene. (B) Each bar shows the percentage of EGFP-positive cells (mean ± SD; n = 3) measured by using FACS. (**p<0.01, compared to the ANAM control group).


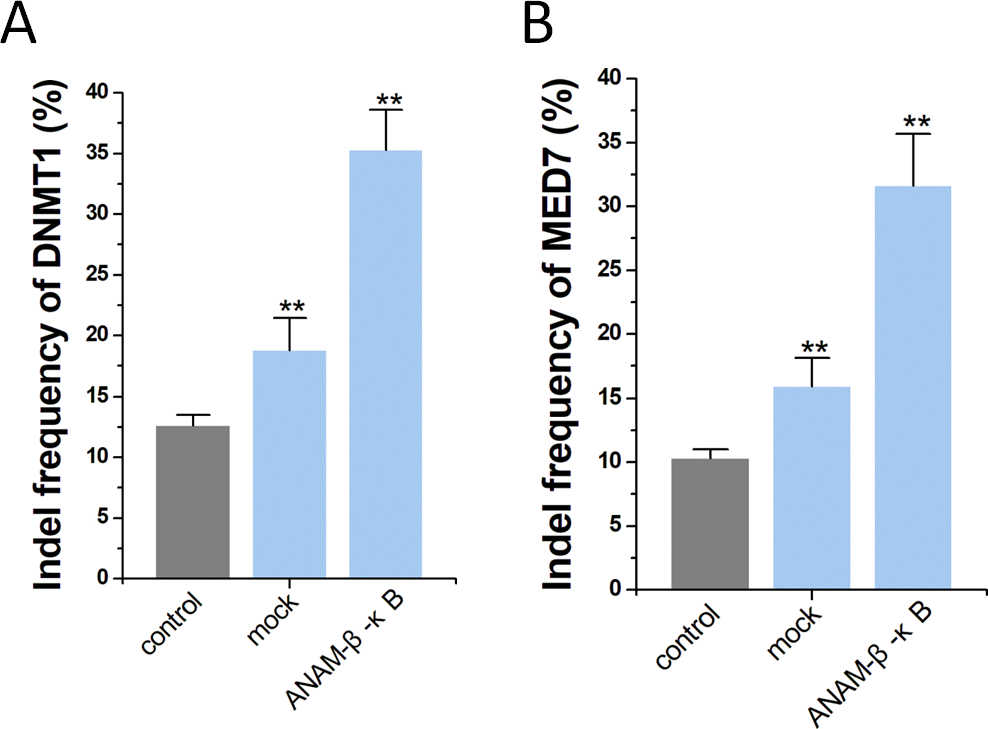


**Supplementary Figure 7**

**Detection of the effect of ANAM on the CRISPR knock-out in human iPS cells.**

The indel frequencies of DNMT1 (A) and MED7 (B) in human iPS cells were shown. Results are shown as mean ± SD. All experiments were repeated three times. (**p<0.01, compared to the ANAM control group).


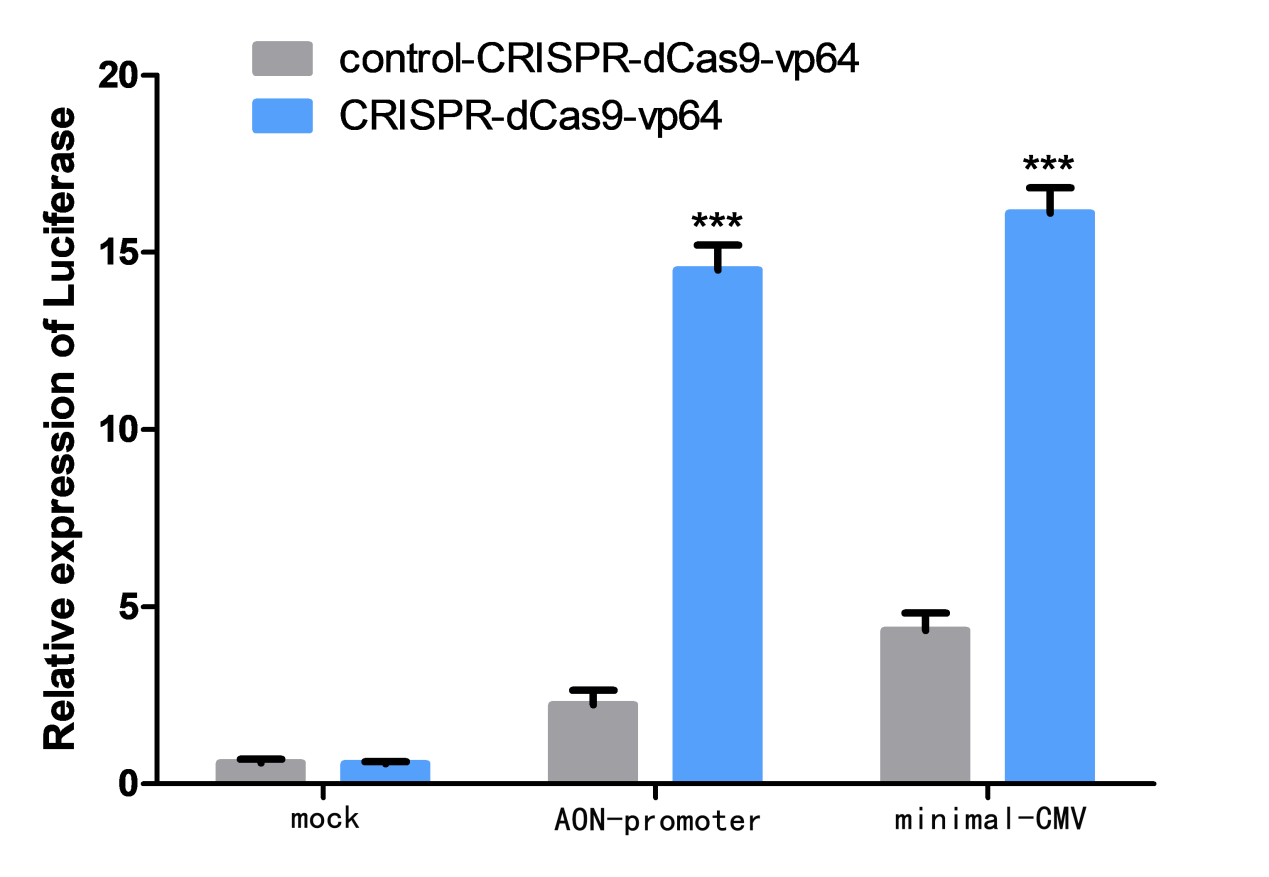


**Supplementary Figure 8**

**All-or-nothing promoter.** The TATA-box is one of the core components of the eukaryotic promoter. It is about -30 bp (-25~-30bp) upstream of most eukaryotic gene transcription initiation sites and consists essentially of A/T base pairs. The choice to determine the initiation of transcription of a gene is one of the binding sites of RNA polymerase. In the initiation of transcription of the mRNA precursor, the transcription factor TF2 and the TATA box are first combined to form a stable complex, and then the other factors bind to the DNA in a certain order to form a transcription initiation complex and start transcription. We first tried using only the TATA box to drive the expression of downstream genes. We placed random nucleic acid sequences upstream and downstream of the TATA-box and activated expression of downstream genes (firefly luciferase) with CRISPR-dCas9-vp64. In the mock group, there is no promoter to drive the expression of the luciferase reporter gene. There is no expression of luciferase with or without the presence of the CRISPR-dCas9-VP64. Comparing to the minimal-CMV group, the AON-promoter group showed better all-or-nothing characteristics. (***<0.001 compared to the mock group). The firefly luciferase activity was normalized to the Renilla luciferase activity (firefly luciferase/Renilla luciferase) and presented as relative luciferase activity.


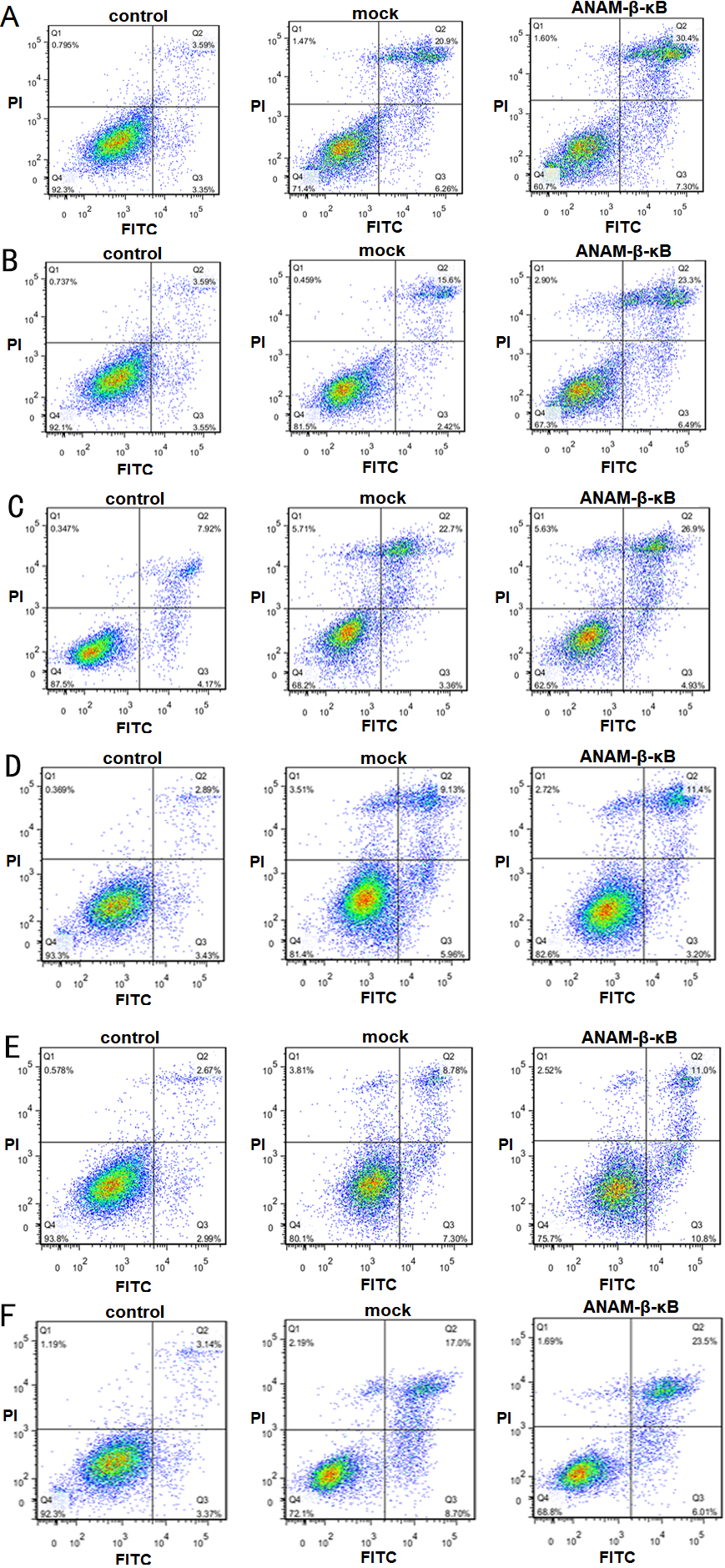


**Supplementary Figure 9**

**The apoptotic flow pattern.** Apoptosis induced by the *Bax* gene in 5637 (A), SW780 (B), and Hela (C) cells was activated by the CRISPR-dCas9-vp64. Apoptosis mediated by the *Bcl-2* gene inhibition in 5637 (D), SW780 (E), and Hela (F) cells was induced by the CRISPR-dCas9.


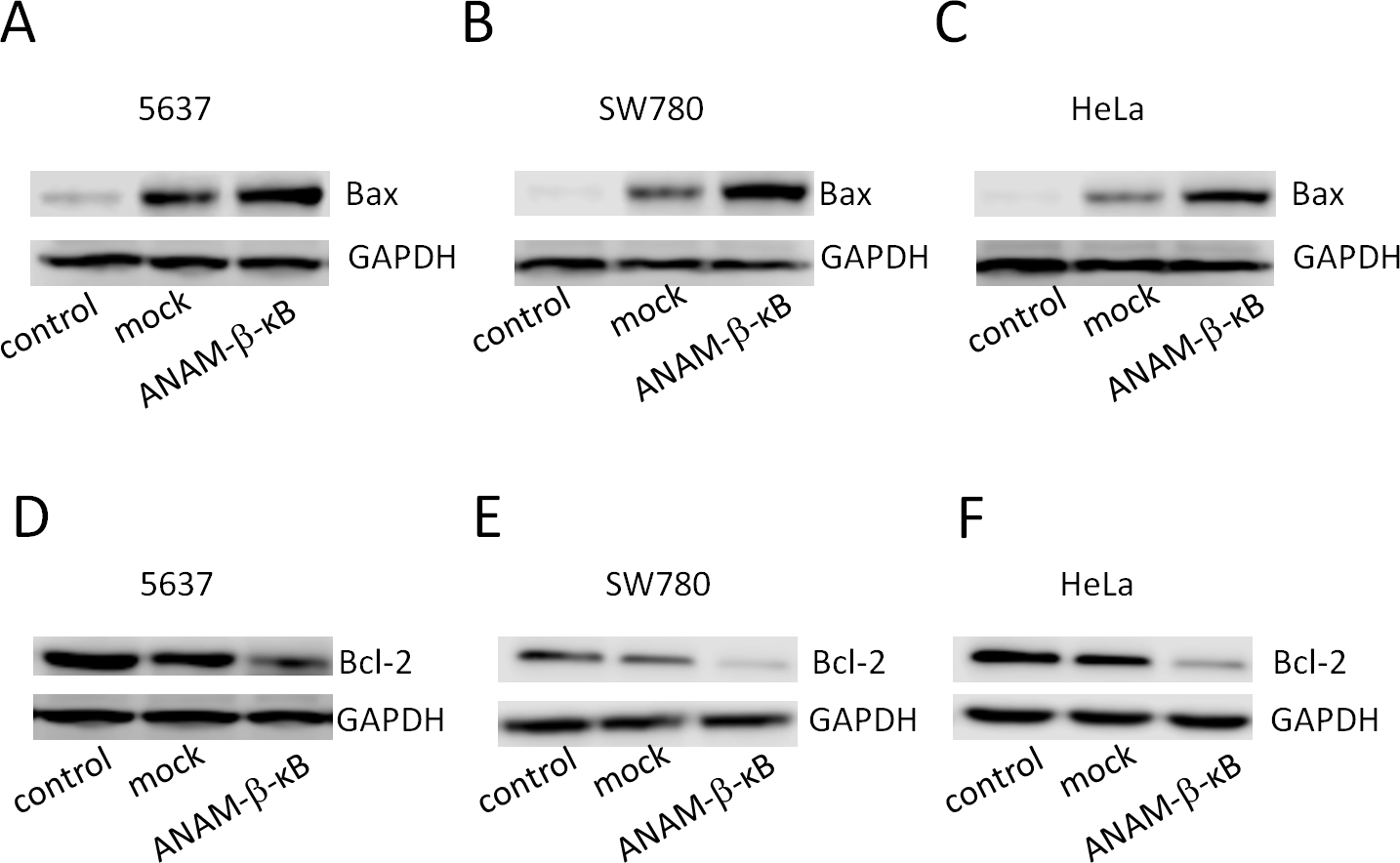


**Supplementary Figure 10**

**ANAM-β-κB improved the activation efficiency of the *Bax and Bcl-2* gene by CRISPR-dCas9-vp64 in cells.** The results of western-blot assay showed that CRISPR-dCas9-vp64 activated the expression of the *Bax* gene in 5637 cells (A), SW780 cells (B) and HeLa cells (C), and that CRISPR-dCas9-KRAB inhibited the expression of the *Bcl-2* gene in 5637 cells (D), SW780 cells (E), and HeLa cells (F).

Supplementary table 1: The sequences of ANAMs in this study.

| name | relative sequences (5′-3′) |
| --- | --- |
| ANAM-β | AGGCCGATCTATGGACGCTATAGGCACACCGGATACTTTAACGATTGGCT |
| ANAM-2β | AGGCCGATCTATGGACGCTATAGGCACACCGGATACTTTAACGATTGGCT**CAACAACAACAACAACAA**AGGCCGATCTATGGACGCTATAGGCACACCGGATACTTTAACGATTGGCT |
| ANAM-3β  ANAM-κB  ANAM-2κB  ANAM-3κB  ANAM-2κB  ANAM-2SκB  ANAM-β-κB | AGGCCGATCTATGGACGCTATAGGCACACCGGATACTTTAACGATTGGCT**CAACAACAACAACAACAA**AGGCCGATCTATGGACGCTATAGGCACACCGGATACTTTAACGATTGGCT**CAACAACAACAACAACAA**AGGCCGATCTATGGACGCTATAGGCACACCGGATACTTTAACGATTGGCT  GCATCCTGAAACTGTTTTAAGGTTGGCCGATGC  GCATCCTGAAACTGTTTTAAGGTTGGCCGATGC**CAACAACAACAACAACAA** GCATCCTGAAACTGTTTTAAGGTTGGCCGATGC  GCATCCTGAAACTGTTTTAAGGTTGGCCGATGC**CAACAACAACAACAACAA**GCATCCTGAAACTGTTTTAAGGTTGGCCGATGC**CAACAACAACAACAACAA**GCATCCTGAAACTGTTTTAAGGTTGGCCGATGC  GGGAGCGGCCGATCTATGGACGCTATAGGCACACCGGATACTTTAACGATTGGCCGCCTGCTGCGGGCCGATCTATGGACGCTATAGGCACACCGGATACTTTAACGATTGGCCCGCAGCAGTTCCC  GGGAGCGCATCCTGAAACTGTTTTAAGGTTGGCCGATGCGCCTGCTGCGGCATCCTGAAACTGTTTTAAGGTTGGCCGATGCCGCAGCAGTTCCC  GGGAGCGGCCGATCTATGGACGCTATAGGCACACCGGATACTTTAACGATTGGCCGCCTGCTGCGGCATCCTGAAACTGTTTTAAGGTTGGCCGATGCCGCAGCAGTTCCC |

Note: The bolded parts are linkers between the elements.

Supplementary table 2: Sequence of primers used in this study.

| name | relative sequences (5′-3′) |
| --- | --- |
| MYC | F: GGCTCCTGGCAAAAGGTCA  R: CTGCGTAGTTGTGCTGATGT |
| Cyclin D1 | F: GCTGCGAAGTGGAAACCATC  R: CCTCCTTCTGCACACATTTGAA |
| TRAF1 | F: TCCTGTGGAAGATCACCAATGT  R: GCAGGCACAACTTGTAGCC |
| BclXL | F: GAGCTGGTGGTTGACTTTCTC  R: TCCATCTCCGATTCAGTCCCT |
| IFN-β | F: ATGACCAACAAGTGTCTCCTCC  R: GGAATCCAAGCAAGTTGTAGCTC |
| TNF-α | F: CCTCTCTCTAATCAGCCCTCTG  R: GAGGACCTGGGAGTAGATGAG |
| IL-12 | F: ACTCACCTCTTCAGAACGAATTG  R: CCATCTTTGGAAGGTTCAGGTTG |
| Bax | F: CCCGAGAGGTCTTTTTCCGAG  R: CCAGCCCATGATGGTTCTGAT |
| Bcl-2  CD23 | F: GGTGGGGTCATGTGTGTGG  R: CGGTTCAGGTACTCAGTCATCC  F: ATGAATCCTCCAAGCCAGGA  R: GCCACAGGAGAAGCAGAGTC |

Supplementary table 3: Sequence of AON-promoter

| name | relative sequences (5′-3′) |
| --- | --- |
| AON-promoter | CGCACTTTGCTGCCTCACGCGC**TATATAA**GCCAGCTTTGACCGCGTACGAACGAGATCGC |
